# High-Resolution Episcopic Imaging for Visualization of Dermal Arteries and Nerves of the Auricular Cymba Conchae in Humans

**DOI:** 10.3389/fnana.2020.00022

**Published:** 2020-05-12

**Authors:** Babak Dabiri, Stefan Kampusch, Stefan H. Geyer, Van Hoang Le, Wolfgang J. Weninger, Jozsef Constantin Széles, Eugenijus Kaniusas

**Affiliations:** ^1^Institute of Electrodynamics, Microwave and Circuit Engineering, Vienna University of Technology, Vienna, Austria; ^2^SzeleSTIM GmbH, Vienna, Austria; ^3^Division of Anatomy, MIC, Medical University of Vienna, Vienna, Austria; ^4^Department for Vascular Surgery, University Clinic for Surgery, Medical University of Vienna, Vienna, Austria

**Keywords:** auricular vagus nerve, cymba conchae, 3D modeling, episcopic imaging, electrical stimulation

## Abstract

Therapeutic applications of auricular vagus nerve stimulation (VNS) have drawn recent attention. Since the targeted stimulation process and parameters depend on the electrode–tissue interaction, the lack of structural anatomical information on innervation and vascularization of the auricle restrain the current optimization of stimulation paradigms. For the first time, we employed high-resolution episcopic imaging (HREM) to generate histologic volume data from donated human cadaver ears. Optimal parameters for specimen preparation were evaluated. Anatomical 3D vascular and nerve structures were reconstructed in one sample of an auricular cymba conchae (CC). The feasibility of HREM to visualize anatomical structures was assessed in that diameters, occupied areas, volumes, and mutual distances between auricular arteries, nerves, and veins were registered. The selected region of CC (3 × 5.5 mm) showed in its cross-sections 21.7 ± 2.7 (mean ± standard deviation) arteries and 14.66 ± 2.74 nerve fibers. Identified nerve diameters were 33.66 ± 21.71 μm, and arteries had diameters in the range of 71.58 ± 80.70 μm. The respective occupied area showed a share of, on average, 2.71% and 0.3% for arteries and nerves, respectively, and similar volume occupancy for arteries and nerves. Inter-centroid minimum distance between arteries and nerves was 274 ± 222 μm. The density of vessels and nerves around a point within CC on a given grid was assessed, showing that 50% of all vessels and nerves were found in a radial distance of 1.6–1.8 mm from any of these points, which is strategically relevant when using stimulation needles in the auricle for excitation of nerves. HREM seems suitable for anatomical studies of the human ear. A 3D model of CC was established in the micrometer scale, which forms the basis for future optimization of the auricular VNS. Obviously, the presented single cadaver study needs to be validated by additional anatomical data on the innervation and vascularization of the auricle.

## Introduction

Auricular vagus nerve stimulation (VNS) is a new neuromodulatory technique for the treatment of, for instance, epilepsy (Bauer et al., 2016), chronic low back pain (Sator-Katzenschlager et al., 2004), and psychiatric disorders like autism (Cimpianu et al., 2017). Moreover, the auricular VNS has some effects on cardiovascular system like suppressing atrial fibrillation (Stavrakis et al., 2015) and improving cardiac function in patients with coronary artery disease (Afanasiev et al., 2016), as summarized in our recent reviews (Kaniusas et al., 2019a,b).

The electrical stimulation of the peripheral branch of the auricular vagus nerve (aVN) within the external ear provides an input to the brain stem and thus a noninvasive possibility to modulate various brain functions (Mercante et al., 2018). The stimulation can be performed either transcutaneously with surface electrodes (Straube et al., 2015) or percutaneously with needle electrodes (pVNS; Zamotrinsky et al., 1997, 2001). While the transcutaneous stimulation yields a rather diffuse stimulation, pVNS is able to target more precisely aVN and requires less current to stimulate aVN fibers (Samoudi et al., 2017). In pVNS, needle electrodes are placed in close vicinity to the visible vessels wired roughly in parallel to aVN, as identified by transillumination (Kaniusas et al., 2010).

Electrodes are placed in and close to aVN regions. In particular, the efficacy of the auricular VNS depends on the proper placement of electrodes in regions innervated by aVN and a valid understanding of the regional anatomy. In particular, the cymba conchae (CC) of the external human ear is of special interest because of its sole vagal innervation (Peuker and Filler, 2002). However, a detailed structural and topological vascularization and innervation of CC is poorly investigated in scientific literature.

The three-dimensional vascularization of the pinna of the external ear was investigated in Tilotta et al. (2009) without the concomitant analysis of innervation. The innervation was visualized in the cavum conchae and the ear channel by detailed histological staining (Bermejo et al., 2017). A complete view on the innervation of the human pinna using standard sectioning methods can be found in Peuker and Filler (2002), the only available anatomical study claiming a sole vagal innervation of CC. However, Burger and Verkuil (2018) showed some inconsistencies with respect to aVN innervation regions, whereas Badran et al. (2018) and Burger and Verkuil (2018) made a controversy on an optimal region for transcutaneous auricular VNS. Another study (Safi et al., 2016) investigated the number of myelinated axons in the main branch of the aVN and categorized them by diameter into Aβ (fibers with the diameter 7–10 μm) and Aδ (fibers with the diameter 2–5 μm), whereas Aβ and Aδ fibers comprised 20% and 50% of the total myelinated aVN axons, respectively. Nonetheless, a complete picture on the innervation, vascularization, and the local anatomy of the pinna of the external ear, especially of CC, is missing.

High-resolution episcopic microscopy (HREM) is a validated tool to assess the local anatomical structure, which allows the generation of micrometer precision digitalized volume datasets (Weninger et al., 2006; Mohun and Weninger, 2011; Geyer and Weninger, 2019). Unspecifically whole-mount stained histological tissue samples with tissue-specific differences provide a reasonable contrast between different structures in stacks of images and allows for visually tracing nerves, arteries, and veins within the generated volume data. Such tracing is crucial to generate better anatomical information of the ears’ innervation and vascularization. Originally developed for researching embryos of biomedical model organisms, HREM has already been used to characterize the arteries of normal human skin (Geyer et al., 2013, 2014; Tinhofer et al., 2018), for analyzing vascularization during wound healing in animal models (Wiedner et al., 2014; Ertl et al., 2018), and even for analyzing the structure of plants and skin replacement material (Geyer et al., 2015; Izhaki et al., 2018).

In this article, HREM is validated as a suitable method to resolve the wiring of vascular and nerve structures in CC, and a detailed analysis is performed within a single human cadaver ear; preliminary data are published in Razlighi et al. (2018). For the first time, geometrical and volume data are provided on arterial and venous vessels, as well as nerves, using statistical methods. These anatomical data can further be used to better optimize stimulation paradigms in the auricular VNS, and especially allow a targeted positioning of stimulation electrodes.

## Materials and Methods

A flowchart of the implemented processing steps is shown in Figure 2.

**Figure 1:**
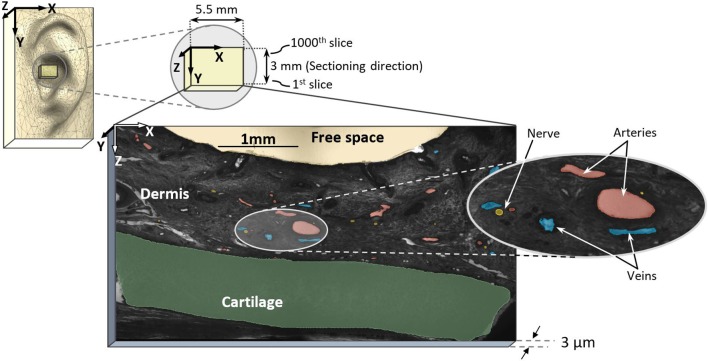
Sampling region in external pinna of the ear and high-resolution episcopic imaging (HREM) image with marked arteries (red), nerves (yellow), and veins (blue).

**Figure 2:**
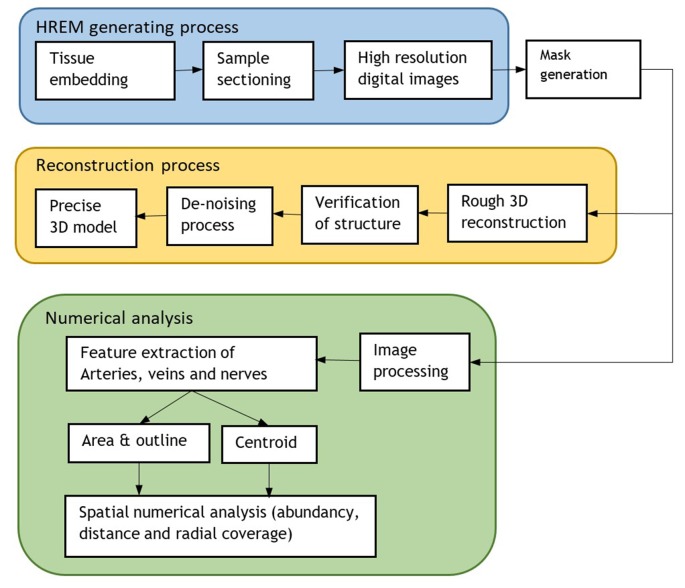
Flowchart of the whole process including the HREM generation for 3D model reconstruction and numerical analysis.

### Preparation of Episcopic Images and Reconstruction

Six body donors, two males (aged 74 and 88 at time of death) and four females (aged 84, 94, 95, and 95 years at time of death) were perfused with 4% carbolic acid and 1% paraformaldehyde for a total of 24 h and then immersed in a mixture of 4.5% carbolic acid and 0.9% paraformaldehyde for 6–12 months. The auricles were collected and the skin of the center of the auricular CC (six left, five right) was dissected and stored in 4% neutral buffered formaldehyde until it was processed for volume data generation. The tissue blocks measured 8 × 7 × 16 mm. Tissue harvesting and processing were performed according to local ethics regulations.

The collected tissues were processed for volume data generation with HREM (Weninger et al., 2006; Mohun and Weninger, 2012a) and several protocols for sample processing were tested (Table 1). The specimens yielding the best data quality (*n* = 3, see Table 1) were rinsed in running tap water for 2 days and then they were dehydrated in a series of ethanols of increasing concentrations [2 h in 30%, 2 h in 50%, 6 days in 70%, 2 h in 80%, 2 h in 90%, and 4 h (two changes) in 96%]. All ethanols contained 0.4 g eosin (Eosin spritlöslich, Waldeck GmbH and Co, KG, Germany) per 100 ml. Protocols tested are summarized in Table 2.

**Table 1:** Age and dimension of related high-resolution episcopic imaging (HREM) datasets.

Dataset No.	Sample (*X* × *Z* × *Y*)*	Image Size (*X* × *Z*)*	Image resolution	No. of images	Age
1	3 × 3 × 12 mm	1,010 × 1,010	3.00 × 3.00 μm	4,000	95
2	5.6 × 3 × 6 mm	3,731 × 2,068	1.48 × 1.48 μm	2,000	95
3	8.5 × 6 × 12 mm	2,886 × 2,058	2.96 × 2.96 μm	4,000	84

*Dimensional coordination is shown in Figure 1.

**Table 2:** Protocols for sample processing to prepare for HREM data generation.

	Washing	30%	50%	70%	80%	90%	96%	Infiltration
Protocol 1	2 days + Eo	2 h + Eo	2 h + Eo	6 days + Eo	2 h + Eo	2 h + Eo	2 × 2 h + Eo	5 days
Protocol 2	2 days	2 h + Eo	2 h + Eo	6 days + Eo	2 h + Eo	2 h + Eo	2 × 2 h + Eo	5 days
Protocol 3	2 days	2 h + Eo	2 h + Eo	Weeks + Eo	16 h	3 h	2 × 3 h	4 days

After dehydration, the specimens were infiltrated for 5 days in solution A of the JB-4 Plus embedding kit, mixed with 1.25 g catalyst (benzoyl peroxide, plasticized) and 0.4 g eosin 100 ml^−1^ (two changes). After infiltration, they were transferred into embedding molds and embedded in eosin-dyed resin following a standardized embedding protocol (Mohun and Weninger, 2012b; Geyer et al., 2017). After block holder insertion, the molds were sealed airproof for at least 2–3 days. The resulting resin blocks were stored under room conditions until they were processed further.

Digital data generation again followed a standard protocol (Weninger et al., 2006; Mohun and Weninger, 2012c). After mounting the blocks in the HREM apparatus, a series of digital images were created, which showed the block surface after subsequently cutting 3-μm-thick sections. The size of the resulting volume datasets is given in Table 1. Prepared digital images (Table 1) were assessed visually and dataset No. 2 of Table 1 was selected for further analysis, which had the best contrast to differentiate anatomical structure. The dataset had a pixel size of 1.48 × 1.48 μm^2^ and a field of view of 5,522 × 3,060 μm^2^.

For data processing, every 5th image slice was used, resulting in a distance of 15 μm between slices with a total of 200 slices (first 1,000 stack of images from dataset No. 2) for reconstruction (in *y*-axis direction, see Figure 1). For each selected image, masks were prepared for elements such as arteries, nerves, veins, cartilage, and dermis, totaling five masks. Masks were first generated by identifying large arteries and nerve fibers according to their histological appearance on single sections. Then, the circumferential course of the nerves and arteries and their branches were manually re-traced and segmented through the dataset, in line with Geyer et al. (2013). Here, the dermis encloses arteries, nerves, and veins while excluding free spaces and cartilage. All masks were verified by an anatomist. Since all identified elements have a hollow tube shape, they were marked as area. In order not to miss elements running perpendicular to the sectioning direction (*Y* axis), we assessed both rough 3D structure and masks (every 15 μm) in parallel to avoid incongruences here. In fact, masks could be generated every 3 μm to allow even more precise identification of profiles of elements.

**Figure 3:**
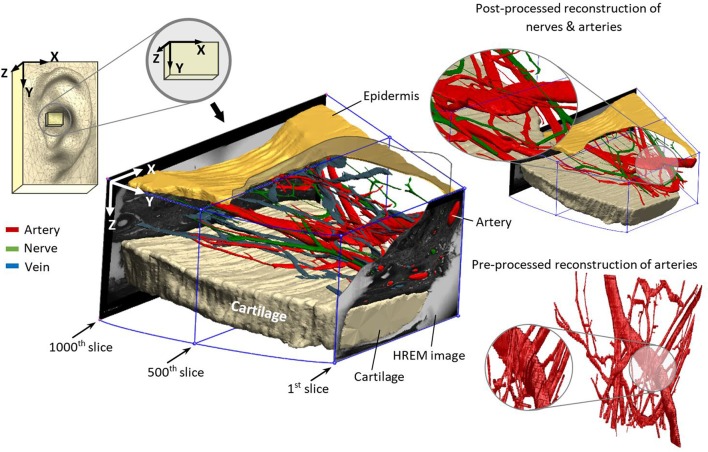
Reconstructed nerves (green), arteries (red), veins (blue), epidermis (mustard), and cartilage (beige) from marked elements in HREM images.

Prepared masks for the different elements were loaded into the software package 3D Slicer (Fedorov et al., 2012) to visually trace the branching points. A 3D surface model was generated by an automated interpolation, which takes into account the branching points of all elements. The scrolling along the different slices facilitated identifying false detection in the mask preparing process. Moreover, 3D Slicer reconstructed a rough 3D model, as shown in Figure 3, which provides spatial tracking of all the elements in the dermis. The generated 3D model has volume data with a single voxel size of 1.48 × 1.48 × 15 μm^3^.

Since the noise and roughness of the generated 3D model (Figure 3) were rather high due to the limited resolution of image pixels (1.48 × 1.48 μm) and the distance in-between consecutive image slices (15 μm), another software package Geomagic Wrap^®^ (3D Systems, Inc., Rock Hill, SC, USA) was used to reduce the noise and post-processing procedures. Finally, for better rendering quality, Geomagic Design^®^ (3D Systems, Inc., Rock Hill, SC, USA) was employed.

### Numerical Data Analysis

The prepared masks of all elements offer structural and geometrical insights. Distances between elements, abundance of each element, or volume occupied by each element was determined. Due to conformational change over histological specimen process on thin-walled veins and consequently occupied area, veins were not processed in numerical analysis. Analyses were based on the centers of mass (centroids) for each type of element. Data are presented as median and/or interquartile range (IQR), supplemented by mean ± standard deviation. All data were processed and analyzed in MATLAB R2019a (The MathWorks Inc., Natick, MA, USA).

#### Abundance

**Figure 4:**
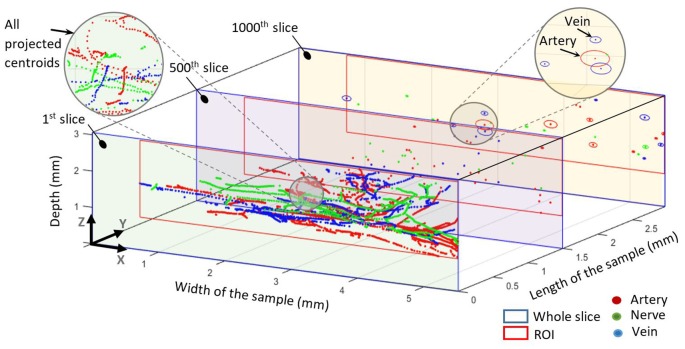
All centroids mapped into the first slice. The 500th slice at 1.5 mm shows all centroids in this slice for all elements. The 1,000th slice at 3 mm shows the centroids and elliptical outlines of each element. The blue outline represents the whole slice whereas the red outline represents the common region of interest for each slice.

Grayscale masks were digitized and processed to extract the spots, which represent the different elements. The intersection of major and minor axes for each spot was assumed as center of mass (centroid). Since we wanted to investigate only arteries and arterioles but not capillaries, we preprocessed generated masks to exclude capillaries. Thus, structures in the artery’s masks with less than 10 μm diameter were eliminated. In order to find out the element’s abundance and the trajectories of each element, we mapped all centroids of all slices into the first slice, as illustrated in Figure 4. The resulting trajectories of centroid patterns demonstrate the conformational structure of the analyzed elements.

#### Occupied Area and Volume

Since we marked the outlines of each element, our masks also contain information on the respective occupied area. For each element, we calculated the occupied percentage area and volume within the dermis. Some elements close to borders, which were not easy to verify along the *Y* axis, were excluded to reduce the false detection. Hence, the region of interest for each slice was defined based on detected components in the corresponding slice. This assured the normalization of areas with real detectable elements and excludes suspicious elements.

#### Distances Between Components

A 3D spatial pattern of detected centroids was reconstructed, which represented the spatial position of nerve and vessel in each slide. Distances were investigated between elements by measuring the special geometrical distance between centroids within individual spatial slides. All mutual distances between elements were assessed, as well as minimum distances between the different elements. Need to emphasize that this distance does not consider the minimum distance including the outer surface of the nerve/vessel and thus states only inter-centroid distance.

#### Radial Coverage of Components

Since the sectioning direction (*Y* axis) shows elements inside the dermis, we reconstructed a virtual 3D structure with known distance between slices. This virtual structure provides access from the *Z* direction to investigate how a generated electric field by percutaneous aVN stimulation (pVNS) and a needle electrode may reach elements. Assuming an inserted electrode needle in *Z* axis generates a cylindrical electric field, we counted each kind of elements by gradually (0.1 mm) increasing the radius of this cylinder. A grid consisting of 17 points was chosen in the *XY* plane for electrode placement to cover the whole sample. Each intersection point of the grid had 1 × 0.5 mm distance for *X* and *Y* axis (see Figure 7 right corner).

**Figure 5:**
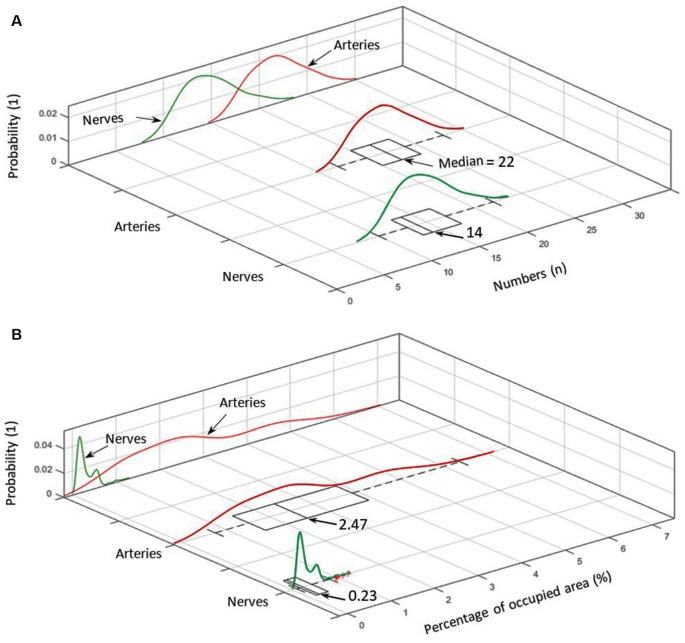
**(A)** Number distribution of elements for arteries (red) and nerves (green) including the corresponding probability density within the analyzed cymba conchae (CC). **(B)** The probability density of the occupied area in percentage for nerves and arteries.

**Figure 6:**
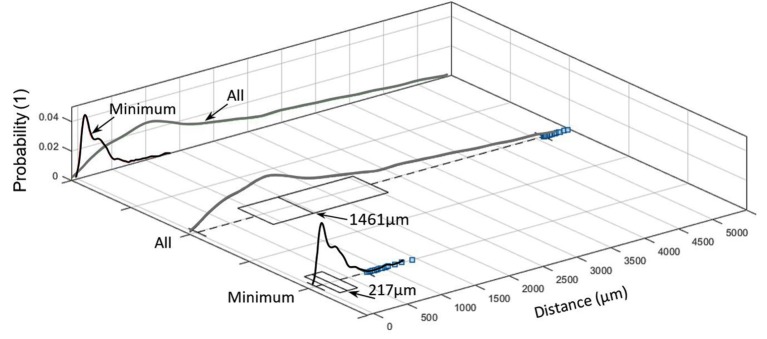
Inter-centroid distance and minimum distance between nerves and arteries within each slide (blue squares denote outliers).

**Figure 7:**
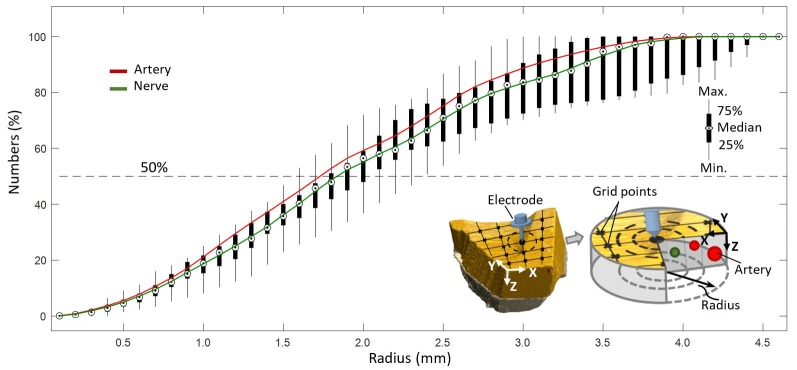
Coverage of arteries and nerves with increasing radius from a point on a predefined grid with resolution of 0.1 mm.

#### Diameter

Due to the preparation process with the partial collapse of vessels and the varying axial orientation of the nerves/vessels with respect to the sectioning direction, the cross-section of each individual element was not geometrically defined. Hence, we fit each observed area to a circular structure in order to estimate its effective diameter. Capillary structures were excluded from further analysis by excluding all arterial structures with diameters smaller than 10 μm.

#### Estimation of Aβ and Aδ Fibers of aVN

Total cross-sectional area of nerve fibers in the analyzed sample from the CC region—with the assumed 100% innervation by aVN—provides some numerical information for the estimation of Aδ and Aβ nerve fiber numbers in aVN. We estimated the number *N*_Aδ_ and *N*_Aβ_ (=*N*_AN_ − *N*_Aδ_) of Aδ and Aβ fibers, respectively, with *N*_AN_ as the total number of fibers, by the following equations:

(1) $$A_{\text{AN}}=(A_{\text{CC}}/A_{\text{S}})\cdot A_{\text{SN}}$$

(2) $$N_{\text{Aδ}}=(50/20)\cdot N_{\text{Aβ}}$$

(3) $$A_{\text{AN}}=N_{\text{Aβ}}\cdot A_{\text{Aβ}}+N_{\text{Aδ}}\cdot A_{\text{Aδ}}$$

Here, *A*_CC_ is the area of the cymba and cavity of conchae (estimated as an elliptical area with a major axis of 20 mm and a minor axis of 15 mm), *A*_S_ is the sample area, *A*_SN_ is the occupied area by nerve in sample, *N*_Aδ_ is the number of Aδ fibers, *N*_Aβ_ is the number of Aβ fibers, *A*_Aβ_ and *A*_Aδ_ are the respective area estimates out of the mean diameter of Aβ (=8.5 μm) and Aδ fibers (=3.5 μm). The ratio 50/20 results from the aforementioned observation that Aβ and Aδ fibers comprise 20% and 50% of the total myelinated aVN axons (Safi et al., 2016).

The whole aforementioned steps are summarized in Figure 2.

## Results

### 3D Reconstruction

The reconstructed 3D model in Figure 3 demonstrates the location of the sample in the ear and the direction of reconstruction based on HREM images. For a better visualization of the dermis (containing nerves, arteries, and veins), cartilage (beige color) and epidermis (mustard color) were added to the model. This step is crucial to revise the resolution of selected slides—in this work every 5th slide—to avoid missing details in between. The wiring pattern between vessels and nerves (Carmeliet and Tessier-Lavigne, 2005) and the effective sectioning resolution (3 μm) prevent missing details. The visual pre-assessment detects parallel profiles to be considered in the following numerical analysis.

### 3D Spatial Distribution

Centroid patterns of all three elements (Figure 4) with different colors were mapped into the first slice of the sample. The blue frame represents the whole HREM image size. Identified borders typically resembled elliptical shapes because of tissue preparation (i.e., the sectioning direction was not always in parallel to the vessels/nerves direction) and almost collapsed vessel walls of the emptied blood vessels. In Figure 4, the shown components in the 1,000th slice (at 3 mm length of the sample) represent the minor and major axis of each detected spot and do not represent their real outline. The pattern of projected centroids in the first slice (at 0 mm length of the sample) simulates the trajectory projection of all elements on the *XZ* plane.

### Numerical Analysis

Figure 5A shows the number distribution of elements in the sample dermis over all slices for arteries 22 (median; 21.7 ± 2.7) and nerves 14 (median; 14.66 ± 2.74), with arteries being by far the most frequent elements. Figure 5A also shows the probability density for each element. Branching of a big artery leads to a local minimum in the probability distribution of occupied area by arteries. With respect to Figure 5B, arteries also occupy the biggest area of 2.47% (median; 2.71 ± 1.36%) in comparison to nerves 0.23% (median; 0.30 ± 0.20%), based on a region of interest for each slice, which encloses all arteries and nerves in the corresponding slice. Likewise, the volume occupied by each element, given a distance of 15 μm between slices, accounts, on average, to 2.71% for arteries and 0.3% for nerves.

Distance between nerves and arteries in the sample was 1,461 μm (median; IQR of 1,700 μm 1,080 ± 1,238 μm), whereas the minimum distance between them was 217 μm (median; IQR of 262 μm; 274 ± 222 μm; see Figure 6).

These values represent only inter-centroid distance but not the perimeter-to-perimeter distance. This is because of the structural alterations during the tissue preparation and the almost collapsed vessel walls of the emptied blood vessels. These deformities make the perimeter-to-perimeter distance sensitive to the morphological changes so that its calculation would require more complicated computation processes.

### Radial Coverage of the Nerves and Arteries

It was evaluated that 50% of all elements, as depicted in Figure 7, were included in a circular region of radius 1.8 mm with respect to a point (0.1 mm resolution) on a predefined grid, representing the hypothetical position of a needle electrode.

### Arteries and Nerve Diameter

**Figure 8:**
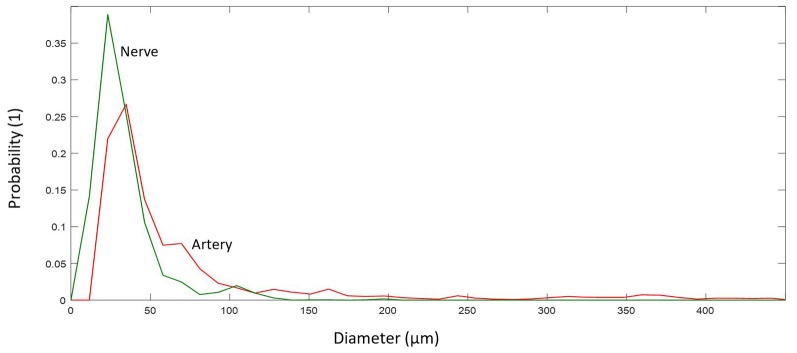
Probability distribution of diameters for different elements.

Arteries and arterioles with a diameter of more than 10 μm showed a larger diameter, 41.38 μm (median; 71.58 ± 80.70 μm), and reside mostly in the range 30–71 μm (IQR), whereas nerves have a smaller diameter, 27.84 μm (median; 33.66 ± 21.71 μm), and reside mostly in the range 20–39 μm (IQR), see Figure 8.

### Estimation of Aβ and Aδ Fibers of aVN

**Figure 9:**
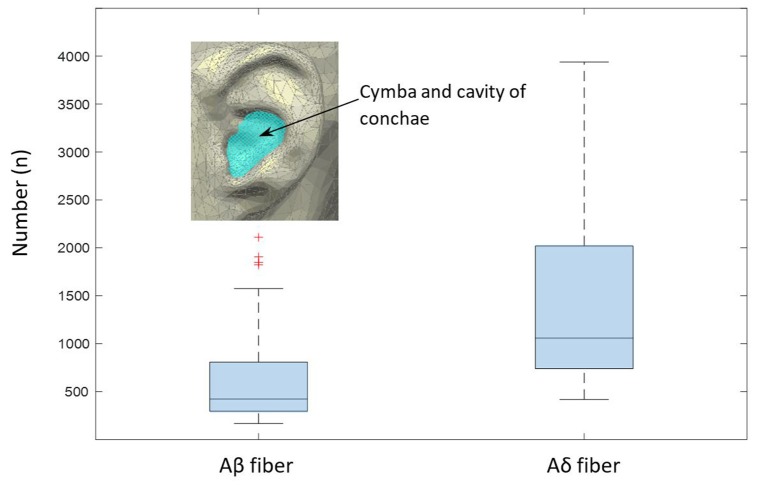
Estimated numbers of myelinated Aβ and Aδ fibers per slide in the cymba and cavity of conchae region, as extrapolated from the analyzed sample (Equation 1). The boxplot shows median values and the associated 25/75 percentiles of the analyzed 200 slides. Whiskers represent outliers.

We estimated number of nerves in the cymba and cavity conchae in Figure 9 based on the formula in the “Materials and Methods” section. The number of Aβ and Aδ fibers was 423 (median; 566 ± 387) and 1,057 (median; 1,417 ± 969), respectively (Figure 9).

## Discussion

The main challenges in the optimization of the auricular VNS are the precise targeting of aVN in the external ear and the selection of proper stimulation parameters to recruit aVN fibers close to the stimulation electrodes (Kaniusas et al., 2019c). The anatomical map of external ear innervation can address the localization of aVN (Peuker and Filler, 2002; He et al., 2012). A targeted selection of stimulated regions in pVNS is performed *via* miniaturized needles located in CC, without involving other auricular nerves. In general, the auricular VNS is performed mostly in CC or tragus. However, there are some inconsistencies with respect to aVN innervation in tragus, while the CC region seems to be solely innervated with aVN.

Furthermore, the lack of deeper knowledge of 3D structure of vessels and nerves—reflecting spatial distribution, abundancy, occupancy percentage, and distance in between individual structures—restricts achieving a realistic stimulation model. Samoudi et al. (2017) showed how the distance between electrode and nerve fibers can affect the percentage of the activated fibers and thus the resulting therapeutic efficiency of pVNS. Unfortunately, their model was simplified to some few main nerves and vessels, which does not necessarily reflect real cases. A more realistic model accounting quantitatively for inner auricular structures would provide comprehensive basis on how to optimize stimulation parameters such as current amplitude, or pulse duration (Samoudi et al., 2017).

For the first time, the present study established a detailed three-dimensional presentation of the innervation and vascularization of the human auricular CC in micrometer precision using HREM. HREM was shown to be a suitable technique to reach high-resolution volume images of this specific auricular region. Most optimal preparation procedures were evaluated to be used in further studies.

In pVNS for portable applications, optimized stimulation paradigms are important not only to avoid unnecessary high current/voltage amplitude and consequently tissue damages but also to avoid high energy consumption. Therefore, having structural information on innervation and vascularization will provide precise and real information for electrode/nerve interaction.

It was found that nerves are emerging roughly in parallel to the local arterial structure, as already elucidated in previous anatomical works (Carmeliet and Tessier-Lavigne, 2005). This parallel wiring is quantitatively shown by the minimum inter-centroid distance of nerves and arteries of 217 μm (median 224 μm). Considering the nerve/vessel distribution diameter with 30–71 and 20–39 μm, respectively, the mean of minimum perimeter-to-perimeter distance would reside between 162 and 192 μm. Furthermore, visual observation of the reconstructed 3D model shows how nerves elongate with arteries and twist in some points (Figure 3). This very detailed investigation supports indirect determination of nerve locations by optical transillumination of the auricle to visualize auricular blood vessels (Kaniusas et al., 2011) for a precise and optimized application of pVNS needle electrodes. Since all detected elements in the studied volume are occupying only 2.7% of the skin, positioning of the electrodes close to vessels seems inevitable for optimal stimulation efficiency.

High sensitivity of axon activation to needle electrode displacement with respect to the axon was already shown to be as high as 15.5% per 0.1-mm electrode displacement in computational studies on pVNS (Samoudi et al., 2017). Further, Samoudi et al. (2017) showed that the effective electric field in a radius of 1.6 mm around the needle electrode is sufficient to activate 100% of a modeled axon population. With respect to the present study, this would mean that 50% of all nerves in the studied volume can be reached, since this is the amount of nerves available in a radius of 1.6–1.8 mm around a point (Figure 7).

It can be hypothesized that nerve fibers visualized by HREM in this study most probably belong to the auricular branch of the vagus nerve (Peuker and Filler, 2002). Although the human auricle is innervated not only by aVN but also by the great auricular nerve, the auriculotemporal nerve, and the lesser occipital nerve (Peuker and Filler, 2002), the innervation probability of the CC solely by aVN is said to be 100%. In future studies, this may be checked in addition to the HREM visualization of a targeted auricular region.

Investigation shows that arteries have double diameter and abundancy in comparison with the nerves. They reside mostly in the range of 30–71 μm diameter, whereas nerves reside between 20 and 39 μm. However, the orientation of the vessel/nerve structure with respect to the sectioning direction would cause uncertainties in the detected cross-section. One solution would be the spatial projection of each cross-sectional area to the perpendicular plane of the inter-centroid lines between each consecutive slide, which would require sophisticated processing algorithms. Favorably, the wiring direction in the analyzed sample was mostly alongside the sectioning direction. Here, a rough 3D reconstruction (Figure 3) was highly reasonable to pre-identify spatial structure of vessels and nerves.

Based on the occupied area by nerve fibers in the given specimen, we estimated the total area occupied by nerves in the cymba and cavity of conchae, like highlighted in Figure 9 (estimated as elliptical area with a major axis of 20 mm and a minor axis of 15 mm). Estimated cross-section of all nerve fibers could be employed to cross check data provided by Safi et al. (2016). Since only the outer borders (epineurium) of the nerves during the mask preparation were marked, a potential overestimation is possible. Based on data in Safi et al. (2016) and Kaniusas et al. (2019b), Aδ fibers have an average diameter of 3.5 μm, and myelinated Aβ fibers have an average diameter and number of 8.5 μm and 370, respectively. Also, they have a relative proportional abundancy of 20/50% (Safi et al., 2016). Therefore, for a known average area of each nerve fiber type (Aβ- and Aδ fibers) and known relative proportional abundancy between them (20/50%), we calculated the estimated number of nerves in the cymba and cavity conchae, based on the scaled cross-sectional area of all nerves in our data. Estimated number for Aβ and Aδ fibers was 423 (median; 566 ± 387) and 1,057 (median; 1417 ± 969), respectively (Figure 9). Moreover, fitting 370 Aβ fibers with an average diameter of 8.5 μm (Safi et al., 2016) into the estimated cross-sectional area of nerves showed 61% (median; 65 ± 34%) occupancy, which qualitatively compares to 20% in Safi et al. (2016).

## Limitations

Our work is the first attempt to use HREM for visualizing the innervation and vascularization of the human CC. No detailed harvesting, specimen processing, and data generation protocols optimized for samples such as the human auricle, composed of cartilage and soft tissues, are yet available. We therefore used different HREM protocol modifications, and though the number of specimens used in this study does not allow for a statistical evaluation, the here-presented protocol represents a useful starting point for further fine-tuning. We finally selected HREM data of a 95-year-old individual for testing the HREM-based data generation approach and the applicability of HREM data for visualization and metric characterization of blood vessels and nerves and simulation of needle placement.

The achieved results demonstrate that the HREM approach can be successfully applied for such analysis. Thus, our work is a sound fundament for starting systematic studies examining larger areas of the auricle in a larger cohort of body donors of different gender and age in order to acknowledge interpersonal variability and site- and age-related changes in tissue morphology and architecture.

Raw HREM images were of sufficiently high contrast to permit precise identification of blood vessels of all dimensions and even distinction of Aδ and Aβ nerve fibers. However, vessel lumina turned out to appear as both empty and blood-filled spaces, and the contrast of nerve fibers is similar to that of surrounding tissue. This makes automated segmentation virtually impossible. We therefore manually outlined the contour of every single blood vessel and nerve in the single images. To increase the speed in creating the binary dataset, we restricted segmentation to every 5th section image and used automated interpolation tools for outlining the segmented structures in between. Thus, despite the fact that distance of subsequent images was 3 μm and their pixel dimensions were 1.48 × 1.48 μm^2^, the binary data effectively comprised voxels of 1.48 × 1.48 × 15 μm^3^. Since capillaries cannot be securely traced in such volume data, we only acknowledged arteries down to the level of arterioles for morphologic, topologic, and metric analysis. Vessels entering the dataset at its lateral borders, which could not be traced back far enough to differentiate between arteries and veins with certainty, were also not included. Obviously, a significant limitation of the work is that only one ear and a relatively small sample area of CC were investigated.

Similar to traditional microscopy, HREM works with fixed, dehydrated, infiltrated, and resin-embedded samples, which are sectioned on a microtome (Mohun and Weninger, 2012a; Geyer et al., 2017). However, in contrast to traditional histology, it does not rely on the processed physical sections and their microscopic evaluation, but on digital images directly captured from subsequently exposed surfaces of the resin blocks during the sectioning process. As a consequence, HREM data are not subjected to non-affine distortions or random staining intensity introduced by the process of section preparation. However, they feature shrinkages introduced during the required tissue fixation and dehydration steps (Mohun and Weninger, 2012b). These artifacts must be considered when interpreting the results of volumetric and planimetric measurements. Furthermore, histological specimen processing causes artificial collapse of thin-walled veins. This hinders their detection and unpredictably alters the volume the venous system occupies in sections. We therefore did not perform volumetrics of the venous system, but restricted our analysis of this system to morphological and topological descriptions.

The HREM data generation is based on unspecific contrasting of the samples. HREM datasets are of near histologic quality and allow for identification and manual segmentation of nerve fibers and arteries. However, this is a time-consuming process. In principle, HREM has also been shown to offer the possibility to detect specifically labeled structures, though this is still in an experimental stage (Weninger et al., 2006). Currently efforts are undertaken to optimize and establish staining protocols allowing for specific labeling. If successful, automatic detection of blood vessels and nerves would enable us to analyze larger volumes in higher detail in a relatively short time span.

In future work, samples should be validated, which consider not only larger CC regions but also different auricles, providing enough data for statistical assessment. Moreover, highly resolved spatial points would allow computation of minimum distances based on perimeters and not only based on inter-centroid distances.

The main aim of this study was to establish protocols and a workflow to evaluate the density of nerves and arteries in the human auricular CC. Obviously, the presented data based on one individual are only exemplary. Further studies are needed for verification and assessment of inter-individual differences.

## Conclusion

HREM seems to be a suitable technique to visualize auricular innervation and vascularization of the human auricular CC. Such anatomical insight can be used to advance stimulation technologies like pVNS and optimize stimulation settings in the future. Most important, a positioning of needle electrodes near auricular vessels seems reasonable to optimize stimulation efficiency.

## Data Availability Statement

The raw data supporting the conclusions of this article will be made available by the authors, without undue reservation, to any qualified researcher.

## Ethics Statement

Ethical review and approval was not required for the study on human participants in accordance with the local legislation and institutional requirements. The patients/participants provided their written informed consent to participate in this study.

## Author Contributions

BD, SK, SG, WW, JS, and EK contributed to the conception and design of the study. BD, SK, and EK wrote the first draft of the manuscript. SG and WW performed data collection. BD, SK, SG, VL, and EK performed data analysis. All authors performed data interpretation. All authors contributed to manuscript revision and read and approved the submitted version.

## Conflict of Interest

EK and SK are employed by SzeleSTIM GmbH. JS receives honoraria from SzeleSTIM GmbH and owns patents in the field of the auricular vagus nerve stimulation. EK, SK, and JS are shareholders of SzeleSTIM GmbH.

The remaining authors declare that the research was conducted in the absence of any commercial or financial relationships that could be construed as a potential conflict of interest.
